# CRISPR/Cas9-Mediated Genome Editing and Mutagenesis of *EcChi4* in *Exopalaemon carinicauda*

**DOI:** 10.1534/g3.116.034082

**Published:** 2016-09-07

**Authors:** Tianshu Gui, Jiquan Zhang, Fengge Song, Yuying Sun, Shijun Xie, Kuijie Yu, Jianhai Xiang

**Affiliations:** * Key Laboratory of Experimental Marine Biology, Institute of Oceanology, Chinese Academy of Sciences, Qingdao 266071, China; † University of Chinese Academy of Sciences, Beijing 100049, China; ‡ Laboratory for Marine Biology and Biotechnology, Qingdao National Laboratory for Marine Science and Technology, Qingdao 266000, China; § College of Marine Life and Fisheries, Huaihai Institute of Technology, Lianyungang 222005, China

**Keywords:** microinjection, *Exopalaemon carinicauda*, CRISPR/Cas9, chitinase

## Abstract

The development of the type II clustered regularly interspaced short palindromic repeats (CRISPR) system has resulted in the revolution of genetic engineering, and this technology has been applied in the genome editing of various species. However, there are no reports about target-specific genome editing in shrimp. In this research, we developed a microinjection method for the ridgetail white prawn *Exopalaemon carinicauda* and successfully applied CRISPR/Cas9 technology to the genome editing of *E. carinicauda*. Through coinjection of mRNA of Cas9 nuclease and gRNA specialized for *E. carinicauda* chitinase 4 (*EcChi4*), shrimps with indel mutations were obtained. Further analysis showed that the mutations could be transmitted to the next generation. This is the first time that site-specific genome editing has been successfully demonstrated in a decapod, and will further contribute to the study of functional genomics in decapods.

Shrimp are widespread all over the world and play a very important role in mariculture. It is a key goal in genetic analysis to identify which genes contribute to specific biological phenotypes and diseases. Although plenty of functional genes of shrimp had been identified and their functions had forecasted through bioinformatics methods, such research in shrimp remains at the gene (real-time polymerase chain reaction [PCR], *in situ* hybridization, and RNAi, *etc*.) and protein levels (recombinant expression, Western blot analysis, immunohistochemistry, protein–protein interactions, and protein–DNA interactions, *etc*.).

In the last few decades, researchers had done a lot of work on the integration of exogenous genes into the shrimp’s genome, including spermatophore-microinjection (Tsai *et al.* 1997; Li and Tsai 2000), electroporation (Powers *et al.* 1995; Tseng *et al.* 2000), and gene gun bombardment (Gendreau *et al.* 1995). However, compared with microinjection, these methods have a plenty of disadvantages, such as, laborious work, low efficiency of integration, and strong randomness of integration. More importantly, it has not been possible to determine where the exogenous gene will locate. Therefore, more efficient genome editing methods, which are site-specific, are essential for further research on the functional genes of shrimp.

Fortunately, recent advances on the clustered regularly interspaced short palindromic repeats (CRISPR) system have made site-specific genome editing quite easy. Since the CRISPR/Cas9 system was first introduced, with the aim of producing site-specific genetic changes in mammalian cells (Cho *et al.* 2013; Cong *et al.* 2013; Jinek *et al.* 2013; Mali *et al.* 2013), it has mediated site-specific genome editing in more than 20 different animals, including *Drosophila melanogaster* (Bassett *et al.* 2013), zebrafish (Chang *et al.* 2013), *Bombyx mori* (Wang *et al.* 2013), and others (Harrison *et al.* 2014). In crustaceans, the CRISPR/Cas9 system also has been used to introduce mutations in *Daphnia magna* (Nakanishi *et al.* 2014) and *Parhyale hawaiensis* (Martin *et al.* 2016).

The exoskeleton of arthropods, made up of chitin and sclerotized proteins, is a rigid scaffold. In order to grow and develop, arthropods have developed a mechanism of molting to replace their old exoskeleton (Merzendorfer and Zimoch 2003). During the molting process, the chitinases play a dominant role in degrading the old cuticles (Elyakova 1972; Funke and Spindler 1987; Buchholz 1989; Watanabe and Kono 1997; Ote *et al.* 2005; Rocha *et al.* 2012). A wide range of chitinase research has been conducted in insects to investigate their structure, function, and regulation (Merzendorfer and Zimoch 2003; Arakane and Muthukrishnan 2010). Some studies of the chitinases in crustations also exist. However, most research has focused on the cloning and characteristic expression patterns of chitinase genes. Recently, RNAi of a chitinase gene was conducted on the salmon louse *Lepeophtheirus salmonis* (Eichner *et al.* 2014, 2015). Knock-down of *LsChi2* by RNAi induced strong downregulation of *LsChi2* expression in the larval stages and resulted in evident changes in the body dimensions, locomotive behavior, and ability to infect fish of salmon lice (Eichner *et al.* 2015).

In our previous research, we purified and characterized two kinds of chitinase (EcChi1 and EcChi2) from the hepatopancreas of *Exopalaemon carinicauda* (Wang *et al.* 2015), which is one of the most important commercial shrimp in China (Li *et al.* 2012). According to the transcriptome data of *E. carinicauda*, the full-length cDNA sequences of *EcChi1* and *EcChi2* were obtained (Wang *et al.* 2015).

In this research, we obtained a new kind of chitinase from *E. carinicauda* and named it as *EcChi4*. In order to clarify the function of *EcChi4*, we tried to knock-out *EcChi4* by CRISPR/Cas9 technology.

## Materials and Methods

### Rearing and hatching of E. carinicauda

The ridgetail white prawn *E. carinicauda* have been cultured in our laboratory for 5 yr. Mature gravid females and mature male shrimps were selected randomly and cultivated in a 180 liter tank containing 100 liter seawater. After spawning, the one-cell stage embryos were collected from the abdomen and transferred to 10 ml sterilized seawater in a petri dish. Before being subjected to microinjection, the one-cell stage embryos were stored at 4° to keep them from developing.

After injection, embryos were put in petri dishes containing sterilized seawater and cultured on a shaker at 100 rpm at room temperature. After 15 d, the shrimp were hatched and the Mysis larvae of *E. carinicauda* were fed with anemia larvae. When the Mysis larvae grew into juvenile prawns, they were fed with bait.

### Preparation of EGFP mRNA

The EGFP sequence was inserted in the multiple cloning site (MCS) between *Eco*RI and *Not*I of the plasmid pIZT/V5-His (Invitrogen) to construct the recombinant plasmid, and renamed pIZ-EGFP; then, the DNA fragment SP6-EGFP-pA for EGFP mRNA synthesis was amplified from the constructed plasmid pIZ-EGFP. The primers were as follows: SP6-EGFP-F (forward): 5′-GCATTTAGGTGACACTATAGAAACAGGCCACCATGGTGAGCAAGGGCGAGGA-3′; SP6-EGFP-R (reverse): 5′-CGCGCTTGAAAGGAGTGTGTA-3′.

The DNA fragment SP6-EGFP-pA was used as the template for *in vitro* transcription, and the capped EGFP mRNA was synthesized using a SP6 mMESSAGE mMACHINE Kit (Ambion). The synthesized EGFP mRNA was then purified and extracted though phenol–chloroform and stored in aliquots at −80°.

### Designation and preparation of gRNA

The gRNA target site of the chitinase gene *EcChi4* was identified by online tool ZiFiT (http://zifit.partners.org/ZiFiT/ChoiceMenu.aspx) (Sander *et al.* 2010).

*In vitro* transcription was performed with the Thermo Scientific TranscriptAid T7 High Yield Transcription Kit. The synthesized gRNA was purified by phenol–chloroform extraction and stored in aliquots at −80°.

### Preparation of Cas9 mRNA

The pCMV-Cas9 vector (Sigma-Aldrich) was linearized by *Xba*I (Takara, Japan) and purified by ethanol precipitation. The purified linearized fragment was used as a template for *in vitro* transcription with a mMESSAGE mMACHINE T7 Ultra Kit (Ambion) to synthesize the capped and poly(A) tailed mRNA of Cas9. The synthesized Cas9 mRNA was purified and extracted though phenol–chloroform, and stored at −80**°**.

### Microinjection of E. carinicauda embryos

Microinjection was carried out using a Warner PLI-100A Pico-Injector microinjector (Warner Instruments) with standardized Femtotip II sterile microcapillaries (Eppendorf, Germany). Embryos were separated using a dissecting needle. After separation, the embryos were injected under a dissecting microscope using a MN-152 micromanipulator (Narishige). All injected mixtures were prepared in water containing 0.05% of the inert dye phenol red. The injection volume was approximately 0.5 nl.

The EGFP mRNA was injected into one-cell stage embryos of *E. carinicauda* at a concentration of 250 ng/µl; Cas9 mRNA at a concentration of 200 ng/µl and gRNA at concentration of 100 ng/µl were coinjected into one-cell stage embryos of *E. carinicauda*.

### Extraction and amplification of genomic DNA

The Genomic DNA of Mysis larvae or juvenile prawns was extracted and the target fragment was amplified using a MightyAmp Genotyping Kit (Takara) according to the manufacturer’s instructions.

The genomic region flanking the CRISPR target site for *EcChi4* was amplified and the product was purified using a Gel Extraction Kit (OMEGA) following the manufacturer’s protocol. The primers used to amplify the target fragment containing the CRISPR target site are as follows: EcChi4-10F (forward): 5′-TACAGGTTAATTACATCAGCCTGA-3′; EcChi4-10R (reverse): 5′-GTTGTTATCCTACCTGATTGAGAT-3′.

### Mutation detection by T7 Endonuclease I (T7EI) assay and Sanger sequencing

The PCR products were subjected to a reannealing process to enable heteroduplex formation: 95° for 10 min, 95–85° ramping at 2°/sec, 85–25° at 0.3°/sec, and holding at 25° for 1 min (Guschin *et al.* 2010). After reannealing, the hybrid product was incubated with T7 Endonuclease I (NEB) for 20 min and analyzed on a 3% agarose gel. The amplified PCR products were isolated and cloned into the pMD19-T Simple Vector (Takara), which was used for Sanger sequencing.

### Data availability

All mentioned tagged lines are available upon request. The authors state that all data necessary for confirming the conclusions presented in the article are represented fully within the article.

## Results

### Structure and characterization of EcChi4

**Figure 1:**
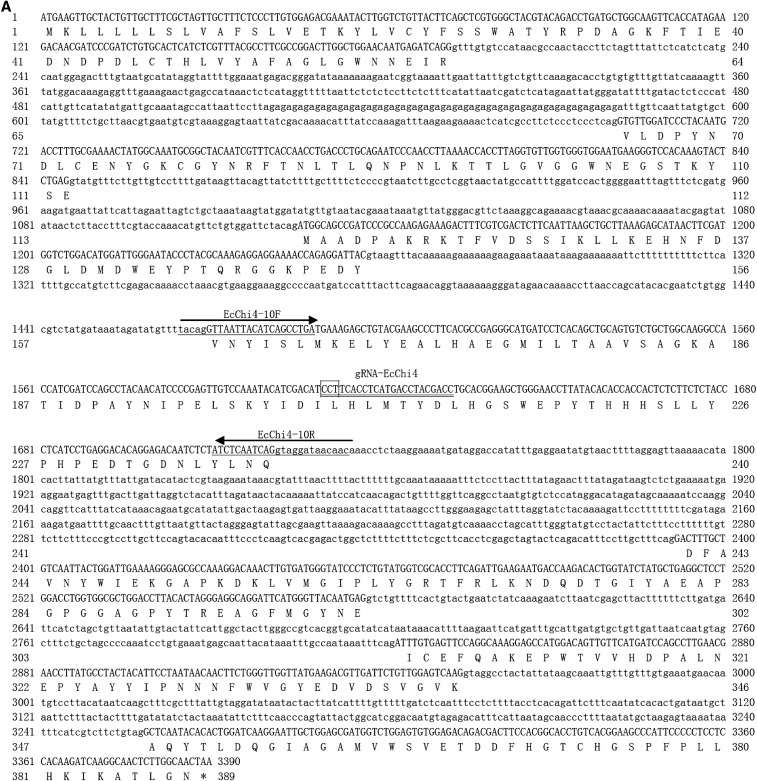

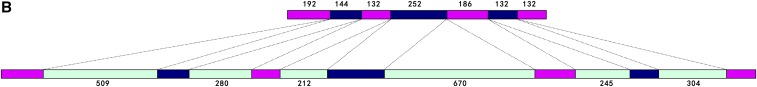
Structure of the *EcChi4* gene. (A) Nucleotide sequence of *EcChi4* gene. Nucleotides are numbered with reference to the translation initiation site (+1). Nucleotides and amino acids are numbered on both sides of the sequence. Uppercase letters indicate exon sequences and lowercase letters represent intron sequences. The gRNA (guide RNA) site is double underlined and the PAM (protospacer adjacent motif) site is indicated by rectangles. The primers used to amplify the CRISPR target site of *EcChi4* are single underlined. (B) Schematic representation of the genomic structures of *EcChi4*. The numbers represent the length of the exon or intron.

Based on the transcriptomic data of *E. carinicauda*, the full-length nucleotide and deduced amino acid sequences of *EcChi4* were obtained (Figure 1A). The open reading frame (ORF) of *EcChi4* encoded 389 amino acids, with a predicted molecular weight of ∼43,849.35 Da and a theoretical isoelectric point (PI) of ∼4.93. The deduced amino acid sequence of EcChi4 contains a signal peptide at position 1–17 and a glycosyl hydrolase family 18 (Glyco_18) domain at position 18–366.

Using the primers (EcChi4-InF and EcChi4-InR), a specific fragment of 3390 bp was amplified from the genomic DNA and then sequenced. The *EcChi4* cDNA sequence showed 100% identity to the corresponding sequence obtained from the genome. Comparison of the genomic DNA sequence with the cDNA sequence of *EcChi4* revealed that there are seven exons and six introns in *EcChi4*. The six introns divide the ORF into seven parts. The sequences at the exon–intron boundaries conformed to the typical eukaryotic splice sites, including an invariant GT at the intron 5′ boundary and an invariant AG at its 3′ boundary (Figure 1A). The organization of the gene is illustrated in Figure 1B.

### Expression of exogenous EGFP mRNA in shrimp embryos

The EGFP mRNA was *in vitro* synthesized using the SP6 polymerase. First, the DNA fragment of SP6-EGFP-pA was cloned from the constructed plasmid pIZ-EGFP, by the primers SP6-EGFP-F/R. The forward primer, SP-EGFP-F, included an upstream spacer (GC), a SP6 promoter (5′-ATTTAGGTGACACTATAGAA-3′), a downstream spacer (5′-ACAG-3′), a Kozak sequence (5′-GCCACC-3′), and a gene-specific sequence for EGFP (5′-ATGGTGAGCAAGGGCGAGGA-3′). The Kozak sequence can enhance the translation initiation of the mRNA (Kozak 1989). The OpIE2 polyadenylation sequence can regulate the transcription termination of mRNA (Theilmann and Stewart 1992). Then, the DNA fragment of SP6-EGFP-pA was used as the template for RNA synthesis. Through the use of the mMESSAGE mMACHINE Kit (Ambion), the synthesized EGFP mRNA gained a 7-methyl guanosine cap structure [m^7^G(5′)ppp(5′)G] at the 5′ end.

**Figure 2:**
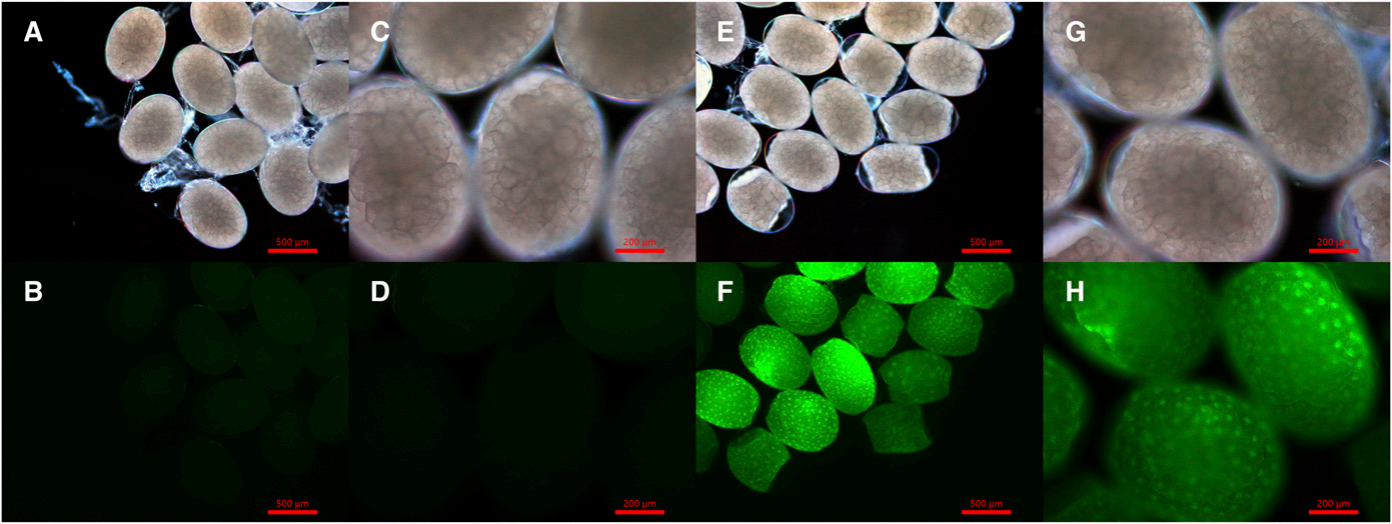
EGFP fluorescence of the injected and control embryos was monitored after 20 hr. (A–D) were the control group; (E–H) were the EGFP-mRNA-injected group. The EGFP-mRNA-injected embryos exhibited specific EGPF fluorescence; whereas the control groups exhibited no EGPF fluorescence. EGFP, enhanced green fluorescent protein; mRNA, messenger RNA.

To test whether the exogenous mRNA could be expressed in the shrimp embryos, the synthesized EGFP mRNA was imported into one-cell stage embryos of *E. carinicauda* through microinjection (as described in the *Materials and Methods*). After 20 hr, the embryos were monitored for EGFP-induced fluorescence under a fluorescence microscope. Compared with embryos of the control group, which were not injected with EGFP mRNA, the mRNA-injected embryos exhibited abundant specific green fluorescence and the fluorescence was visible in almost all the injected embryos (Figure 2). This result indicated that the exogenous mRNA could be expressed in shrimp embryos.

### EcChi4 disruption by the CRISPR/Cas9 system

Based on the result that the exogenous mRNA could be expressed in the shrimp embryos, the Cas9 mRNA and gRNA specific for *EcChi4* were coinjected into one-cell stage embryos (as described in the *Materials and Methods*), to see if CRISPR/Cas9 could work on *E. carinicauda*.

**Figure 3:**
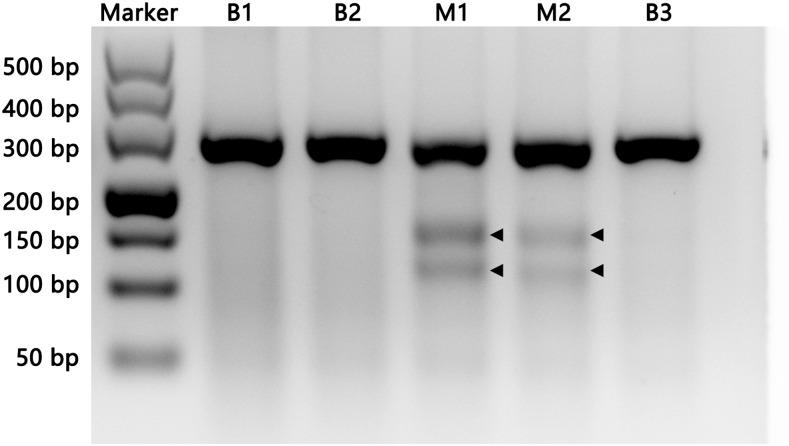
CRISPR/Cas9-induced indels in the *EcChi4* locus. Representative T7 Endonuclease I (T7EI) assay showing CRISPR (clustered regularly interspaced short palindromic repeats)/Cas9-mediated cleavage in single embryos. In five of the injected embryos, two embryos (M1 and M2) were detected with indels. Black arrowheads indicate cleavage bands.

**Figure 4:**
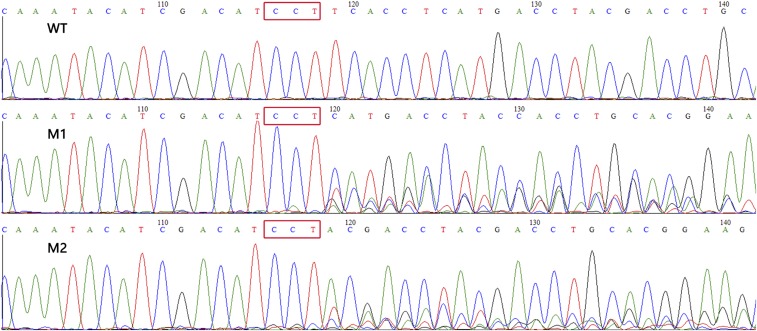
Sanger sequencing of PCR products indicating the indel mutations caused by the Cas9 mRNA and gRNA. The red rectangles represent the PAM site. WT means the wild type, corresponding to lanes B1, B2, and B3 in Figure 2. M1 and M2 similarly correspond to the relevant lanes in Figure 2. gRNA, guide RNA; mRNA, messenger RNA; PAM, protospacer adjacent motif; PCR, polymerase chain reaction.

After 15 d hatching, the Mysis larvae of *E. carinicauda* were collected and used for genomic DNA extraction and amplification of the target fragment through PCR. Then, the T7EI assay was conducted to detect the mutations on the fragment (as described in the *Materials and Methods*). As shown in Figure 3, part of the target fragment DNA was cleaved by T7EI. The result indicated that there were indel mutations in the genomic DNA, which were induced by the Cas9 mRNA and gRNA. To further confirm this result, Sanger sequencing was used to check the indel mutations. DNA sequencing results for the PCR products from F0 shrimps revealed multiple peaks around the Cas9 cut site, consistent with the PCR products being a mixture carrying different indel mutations induced by Cas9/gRNA in the corresponding targeted locus (Figure 4).

**Figure 5:**
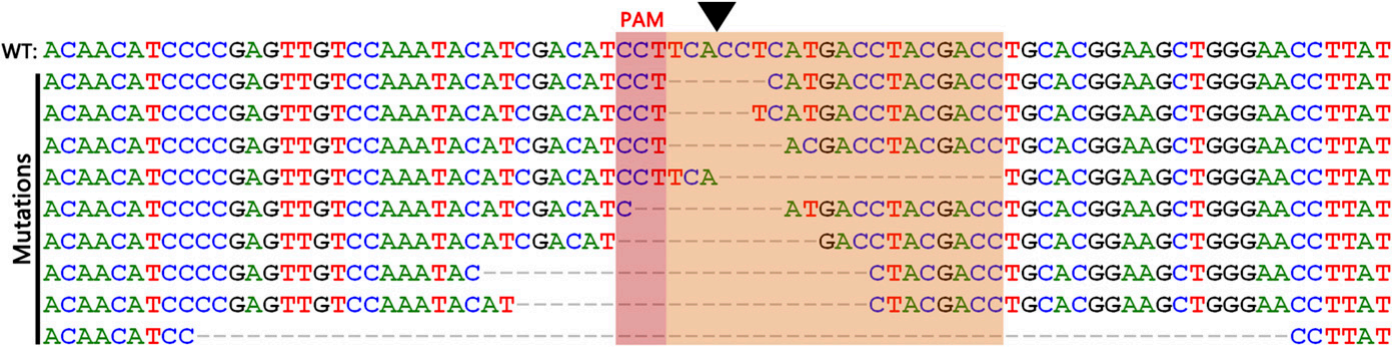
*EcChi4* fragments were amplified and cloned into the pMD19-T simple vector from an individual embryo for sequencing. The brown rectangle is the PAM sequence. The black arrowhead is the cleavage site. The yellow rectangle is the 20 bp genomic target site. Deletion is represented by a dashed line. PAM, protospacer adjacent motif; WT, wild-type.

After confirmation by the T7EI assay and sequencing of the indel mutations, we further identified the mutations on the target site by cloning and Sanger sequencing (Figure 5). Each line in the alignment represents a sequence; the first sequence is the wild-type sequence, the subsequent sequences are the individual mutant clones. Taken together, the results demonstrate that the CRISPR/Cas9 system can mediate mutagenesis in *E. carinicauda* and that mosaic shrimps had been successfully obtained.

### The mutation rate of the CRISPR/Cas9 system injected embryos

The survival and mutation rates of the embryos injected with Cas9 mRNA and gRNA were analyzed (Table 1). In the 247 injected one-cell stage embryos, 88 embryos were hatched and the hatchability was 35.63%; 35 embryos could develop to postlarvae and the reproductive survival rate was 14.17%. Regarding the detection of the mutation for the 35 surviving postlarvae, the number of mutant shrimp was 18 and the mutant rate reached 7.29%. This result indicates that CRISPR/Cas9-induced mutagenesis in *E. carinicauda* is highly efficient and that the survival rate of the injected embryos is quite high.

**Table 1:** Mutation frequencies induced by microinjection of Cas9 mRNA and gRNA

RNA Concentration		Injected Embryos	Hatched Zoea Stage	Survival Postlarvae	Mutant Postlarvae	Survival Rate	Mutant Rate
100 ng/µl (gRNA-*EcChi4*)	200 ng/µl (pCMV-Cas9)	247	88	35	18	14.17%	7.29%

gRNA, guide RNA.

After a further 50 d, the injected embryos developed to adult *E. carinicauda* and their weight and length were statistically analyzed (Table 2). As the data show, the average weight and length of the mutant shrimp are slightly higher those of the control shrimp. However, there is no significant difference between them when these data are assessed using single factor analysis of variance. In addition, no significant morphological change in the mutants was observed. Thus, we speculated that the mutations on the target site of *EcChi4* do not have significant influence on the development and growth of *E. carinicauda*.

**Table 2:** Weight and length of mutant and control shrimp

Number	Mutants		Controls	
	Weight (g)	Length (cm)	Weight (g)	Length (cm)
1	0.17	2.2	0.34	3.0
2	0.28	2.8	0.26	2.4
3	0.24	2.7	0.22	2.6
4	0.08	1.8	0.10	2.1
5	0.11	2.0	0.09	2.0
6	0.28	2.7	0.31	2.9
7	0.30	2.9	0.11	1.9
8	0.18	2.5	0.20	2.5
9	0.23	2.6	0.25	2.7
10	0.38	3.0	0.29	2.8
11	0.63	3.4	0.11	2.0
12	0.10	1.8	0.12	2.0
13			0.21	2.5
14			0.30	3.0
15			0.21	2.5
Means	0.25	2.5	0.21	2.5

### Transmission of mutations to subsequent generations

**Figure 6:**
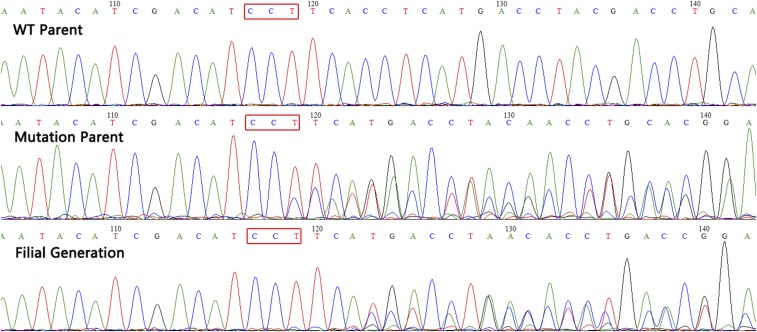
Sanger sequencing of the PCR products of the parental and filial generations. The red rectangles represent the PAM site.

**Figure 7:**
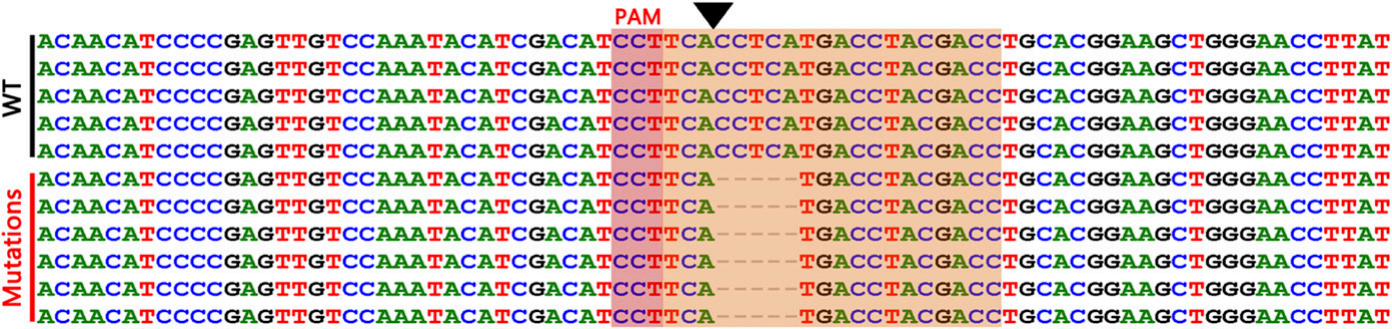
*EcChi4* fragments were amplified and cloned into the pMD19-T simple vector from the filial generations for sequencing. The brown rectangle is the PAM sequence. The black arrowhead is the cleavage site. The yellow rectangle is the 20 bp genomic target site. Deletion is represented by a dashed line. PAM, protospacer adjacent motif; WT, wild-type.

In order to investigate whether the mutations could be transmitted to the next generation, four mutant shrimps were crossed with four wild-type shrimps to produce the filial generations (G1). Through Sanger sequencing, the genotypes of parents and subsequent generations were mapped. As the result show, the wild-type parent was homozygous, the mutant parent was heterozygous, and half of the filial generations were heterozygous (Figure 6). Further identification showed the mutation of the heterozygous progeny from the same family, with one wild-type allele and one allele harboring 5 bp deletions (as described in the *Materials and Methods*) (Figure 7). Those heterozygous progeny (G1) from the same family were then crossed to produce the filial generations (G2). Of 30 sequenced G2 postlarvae, 8 were wild-type, 16 were heterozygous, and 6 were homozygous mutants. The ratio of three genotypes was 1∶2∶1, indicating Mendelian inheritance of the mutant. The results indicate that CRISPR/Cas9-mediated mutagenesis can be generated within the germline and that the mutations can be transmitted to the offspring in *E. carinicauda*. There is no significant morphological change among wild-type, heterozygous, and homozygous mutants of G2 postlarvae at the *EcChi4* locus. In addition, the 5 bp deletions in exon 4 of *EcChi4* cause a shift in the reading frame of EcChi4. Therefore, we conclude that the mutation of *EcChi4* does not influence to the development and growth of *E. carinicauda*.

## Discussion

In this research, we showed four major findings: (1) that the microinjection method was successfully applied to the embryos of *E. carinicauda*; (2) exogenous mRNA was successfully expressed in shrimp embryos; (3) the CRISPR/Cas9 system efficiently generated double-strand breaks (∼50%) to induce a wide range of indels at the *EcChi4* locus in the genome of *E. carinicauda*; and (4) that heritable genome editing can be conducted in *E. carinicauda* via the CRISPR/Cas9 system.

At present, RNAi remains instrumental and universal in decapod research (Sagi *et al.* 2013). However, it involves the injection of adult decapods. The construction of the microinjection system in shrimp breaks down the barrier to the research of functional genes in decapods. Using the microinjection system, a wide range of RNAi knock-down studies on embryos of decapods can be conducted and will accelerate the basic research of decapod animals.

More importantly, this system can be used as a genome editing tool for other decapods. Up to now, genome editing has only been conducted in several crustaceans. In the amphipod crustacean *Parhyale hawaiensis*, the transposable element *Minos* was used to produce transgenic lines (Pavlopoulos and Averof 2005). In the cladoceran crustaceans *D. magna* and *D. pulex*, TALEN and CRISPR/Cas9 were used to mediate targeted mutagenesis (Hiruta *et al.* 2014; Nakanishi *et al.* 2014; Naitou *et al.* 2015). However, this study represents the first time that gene editing has been realized on a decapod crustacean. Using the CRISPR/Cas9 system, 18 *EcChi4* locus mutants of the ridgetail white prawn were established. By crossing the mutant shrimp with the wild-type shrimp, we also got the mutant offspring. Since whole genome data for *E. carinicauda* is not currently available, the evaluation of the off-target effects cannot be conducted. Nonetheless, no morphological change in the 18 mutants and their offspring was observed.

Through the CRISPR/Cas9 system, we have knocked-out a specific gene in shrimp for the first time. This represents a great advance for the research of functional genes in shrimp. It will also provide a knock-in technology approach that can integrate exogenous genes into the genome of *E. carinicauda*. Thus, the CRISPR/Cas9 system is an efficient tool for the genome editing of shrimp that can be used in both scientific research and breeding improvement in aquaculture. In addition, the ridgetail white prawn *E. carinicauda* may be used as a novel model organism for decapod crustaceans to reveal the function of genes relevant to their development, growth, metabolism, and reproduction *in vivo*.
